# The effects of pigment epithelium-derived factor on atherosclerosis: putative mechanisms of the process

**DOI:** 10.1186/s12944-018-0889-z

**Published:** 2018-10-17

**Authors:** Shouyuan Ma, Shuxia Wang, Man Li, Yan Zhang, Ping Zhu

**Affiliations:** 1 Department of Geriatric Cardiology, Chinese PLA General Hospital, Beijing, 100853 China; 2 Department of Cadre Clinic, Chinese PLA General Hospital, Beijing, 100853 China

**Keywords:** Pigment epithelium-derived factor, Atherosclerosis, Coronary artery disease

## Abstract

Cardiovascular disease (CVD) is a leading cause of death worldwide. Atherosclerosis is believed to be the major cause of CVD, characterized by atherosclerotic lesion formation and plaque disruption. Although remarkable advances in understanding the mechanisms of atherosclerosis have been made, the application of these theories is still limited in the prevention and treatment of atherosclerosis. Therefore, novel and effective strategies to treat high-risk patients with atherosclerosis require further development. Pigment epithelium-derived factor (PEDF), a glycoprotein with anti-inflammatory, anti-oxidant, anti-angiogenic, anti-thrombotic and anti-tumorigenic properties, is of considerable interest in the prevention of atherosclerosis. Accumulating research has suggested that PEDF exerts beneficial effects on atherosclerotic lesions and CVD patients. Our group, along with colleagues, has demonstrated that PEDF may be associated with acute coronary syndrome (ACS), and that the polymorphisms of rs8075977 of PEDF are correlated with coronary artery disease (CAD). Moreover, we have explored the anti-atherosclerosis mechanisms of PEDF, showing that oxidized-low density lipoprotein (ox-LDL) reduced PEDF concentrations through the upregulation of reactive oxygen species (ROS), and that D-4F can protect endothelial cells against ox-LDL-induced injury by preventing the downregulation of PEDF. Additionally, PEDF might alleviate endothelial injury by inhibiting the Wnt/β-catenin pathway. These data suggest that PEDF may be a novel therapeutic target for the treatment of atherosclerosis. In this review, we will summarize the role of PEDF in the development of atherosclerosis, focusing on endothelial dysfunction, inflammation, oxidative stress, angiogenesis and cell proliferation. We will also discuss its promising therapeutic implications for atherosclerosis.

## Background

Cardiovascular disease (CVD) is responsible for the majority of morbidity and mortality worldwide, accounting for approximately one third of deaths globally [1, 2]. Atherosclerosis and its cardiovascular ischemic complications are thought to be the major underlying cause of CVD, acute coronary syndrome (ACS) and stroke [3, 4]. Atherosclerosis is a chronic vascular disease with multiple factors and links and is characterized by atherosclerotic lesion disruption with superimposed thrombus formation [5]. This type of vascular pathology is chiefly involved in a combination of endothelial dysfunction, extensive lipid deposition in the intima, enhanced immune responses, proliferation and migration of vascular smooth muscle cells (VSMCs), remodeling of the extracellular matrix (ECM), formation of fibrous caps, atherosclerotic plaque formation, plaque expansion and rupture, and thrombosis of the affected vessel [3]. Atherosclerosis primarily affects the medium and large arteries and causes serious, harmful cardiovascular ischemic events. Accumulated evidence has shown that the atherosclerotic process is typically initiated by endothelial dysfunction [5, 6]. The infiltration of inflammatory cells and release of inflammatory mediators, oxidative stress, cell proliferation and vascular remodeling all play crucial roles in atherosclerosis [7–9]. These processes are the key mechanisms in the development of atherosclerosis [7].

Pigment epithelium-derived factor (PEDF) is of considerable interest due to its anti-inflammatory, anti-oxidant, anti-angiogenic, anti-thrombotic, anti-tumorigenic, neurotrophic and neuroprotective properties [10, 11]. PEDF has been shown to be pleiotropic and was first extracted from the medium of human fetal retinal pigment epithelium (RPE) [12]. It belongs to the serine protease inhibitor (SERPIN) supergene family, located on chromosome 17p13.1 [13]. A great deal of research has been carried out to elucidate the role of PEDF in cardiovascular physiology and pathophysiology, such as in ischemic heart disease, atherosclerosis and ACS [10]. It has been suggested that, along with brain natriuretic peptide (BNP), PEDF concentrations may be a valuable marker for heart failure (HF) prognosis [14]. In addition, PEDF plasma concentrations were closely associated with blood pressure and could predict incident hypertension [15]. The substitution or upregulation of PEDF through the use of medication may prevent and treat occlusive thrombus [16]. Furthermore, Yamagishi S and his colleagues [17] have reported that PEDF could suppress NADPH oxidase-mediated reactive oxygen species (ROS) generation and may play an important role in the development and progression of atherosclerosis. Importantly, serum level of PEDF is believed to be a marker of atherosclerosis in humans [18]. Additionally, our group has carried out recent studies on PEDF, ranging from clinical issues to molecular mechanisms. Initially, we found that plasma PEDF level was significantly lower in ACS patients, and lower PEDF level was associated with adverse cardiac outcomes after ACS [19]. Furthermore, our data showed that plasma PEDF level was significantly lower in CAD patients and PEDF may be used as a potential predicator for coronary severity [20]. An analysis of single nucleotide polymorphisms (SNPs) suggested that the T allele of rs8075977 in the 5′-flanking region of the PEDF gene may be protective for coronary artery disease (CAD) [21]. For these reasons, we have undertaken further investigations. One of the key factors of atherosclerosis, oxidized-low density lipoprotein (ox-LDL), was shown to reduce PEDF through the upregulation of ROS [22]. D-4F can protect endothelial cells (ECs) against ox-LDL-induced injury by preventing the downregulation of PEDF [23]. Moreover, our group found that PEDF might alleviate endothelial injury by inhibiting the Wnt/β-catenin pathway [24]. Therefore, the effects of PEDF have gained much importance in their relation to the major processes in atherosclerosis, such as endothelial dysfunction, inflammation, and oxidative stress, among others. In this review, the current understanding of PEDF will be described and its role in atherosclerosis and pivotal pathophysiologic activities will be highlighted.

## Overview of PEDF

**Fig. 1:**
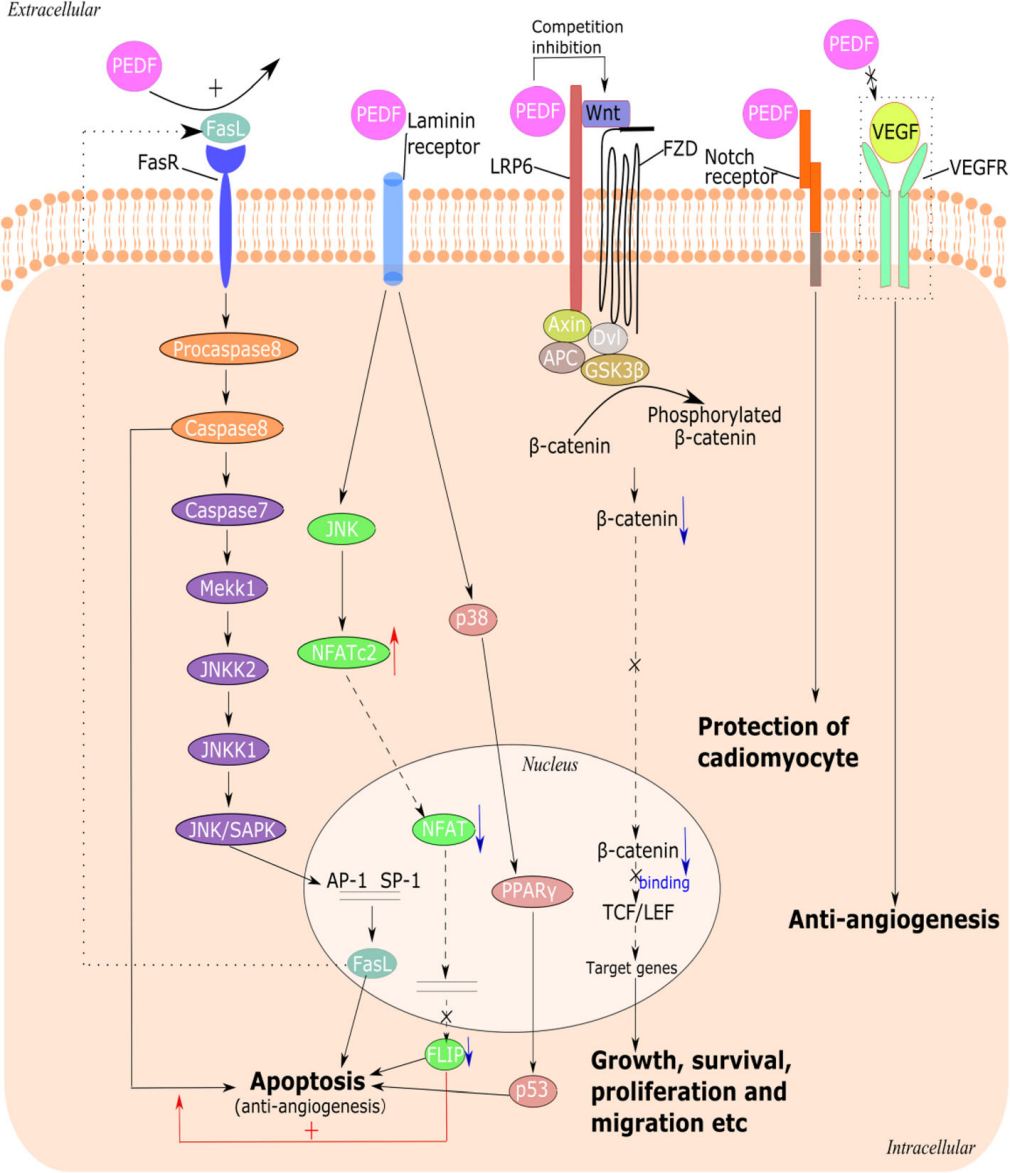
Several common PEDF receptors and downstream pathways in cell. PEDF can upregulate the pro-apoptotic FasL, promoting the binding of FasL to FasR and activation of caspase 8 that induces the cell death cascade under certain conditions. PEDF is a ligand of LR and PEDF-LR complex triggers JNKs. Activated JNKs with higher binding affinity to NFATc2 lead to reduced NFAT in the nucleus, downregulating the anti-apoptotic factor FLIP. Besides, PEDF activates p38 through LR and subsequently result in activation of PPAR-γ. As a result, PEDF causes apoptosis, suggesting its anti-angiogenesis. PEDF is an endogenous antagonist of LRP6, the co-receptor of Wnt/β-catenin pathway. So PEDF can block the pathway, attenuate β-catenin nuclear translocation and inhibit the activity of transcription factor TCF/LEF, suggesting a novel mechanism for its protective effects against diabetic retinopathy and endothelial damage. Other common receptors for PEDF are Notch receptor and VEGFR. The binding of PEDF to Notch receptor plays a key role in the protection of cariomyocyte. PEDF inhibits VEGF-driven angiogenesis through the regulated intracellular proteolysis of VEGFR. PEDF, pigment epithelium-derived factor; FasL, Fas ligand; FasR, Fas receptor; LR, laminin receptor; JNK, JUN N-terminal kinase; NFATc2, nuclear factor of activated T-cells, cytoplasmic 2; NFAT, nuclear factor of activated T-cells; FLIP, FLICE-like inhibitory protein; PPAR-γ, peroxisome proliferator-activated receptor γ; LRP6, low density lipoprotein related protein 6; TCF, T cell-specific transcription factor; LEF, lymphoid enhancer-binding factor; VEGFR, vascular endothelial growth factor receptor; VEGF, vascular endothelial growth factor

PEDF is a 50 kDa secreted, pleiotropic glycoprotein. This protein shares sequence and structural homology with SERPIN family proteins but does not inhibit proteases [25]. Many organs in the human body naturally express PEDF, such as the eye, liver [26], brain [27], pancreas [28], bone [29], adipose tissue and spinal cord [29]. Additionally, PEDF is present in the human heart [30, 31]. As an endogenously produced, extracellular, diffusible and circulating glycoprotein, PEDF is secreted by many cell types in addition to RPE, including endothelial cells [32], cardiomyocytes, fibroblasts [30], macrophages [33] and adipocytes [34]. Nevertheless, there is no clear evidence suggesting which kind of cell or tissue specifically expresses PEDF. Current evidence indicates that PEDF binds to laminin receptor (LR) [35], low density lipoprotein related protein 6 (LRP6) [36], Notch receptor [37], phospholipase A_2_ (PLA_2_) receptor and adipose triglyceride lipase (ATGL) receptor [38]. PEDF has been shown to cause anti-angiogenic, anti-inflammatory and anti-thrombogenic reactions in myeloma cells through the interaction with LR. Accordingly, LR agonist or antagonist may be a target of the reactions in which PEDF is involved. PEDF is an endogenous antagonist of LRP6 and inhibits the canonical Wnt signal pathway [36]. The anti-angiogenic property of PEDF is considered to be dependent upon the induction of Fas ligand (FasL) and Fas receptor (FasR), and result in apoptosis [39]. Besides, the molecular links between PEDF and the PLA_2_ receptor may facilitate cardiomyocyte apoptosis via the Fas apoptotic pathway. The combination of interactions with different receptors may drive different responses, which may be the pathophysiological basis of the abovementioned PEDF properties (Fig. 1).

In addition to certain organs, tissues and cell types, human serum also contains PEDF that is closely associated with several diseases, particularly CVD. Yamagishi reported that the concentration of PEDF in human blood is approximately 100-200 nmol/L, and elevated serum PEDF level may be a counter-system in metabolic syndrome (MetS) [40]. There is statistically significant evidence that the level of PEDF decreased after sustained weight loss, indicating that PEDF may act as a buffer against fat mass [41]. One study has shown that patients with MetS had significantly higher level of serum PEDF than non-MetS subjects; similarly, patients with CAD also had significantly higher serum PEDF level than the non-CAD group. Increased PEDF may act as a protective response against vascular damage and subsequent CAD [42], and there is convincing evidence suggesting that the risk of a clinical event independently and gradually elevates with increasing concentrations of PEDF in patients with advanced HF [14]. ACS patients, however, had decreased plasma and intraplatelet level of PEDF [16]. Meanwhile, we have previously shown that ACS patients had notably lower plasma PEDF level relative to the control group and lower PEDF level was further related to adverse cardiac outcomes after ACS [19], and that PEDF level remarkably decreased in CAD patients compared to the controls [20]. Therefore, it is not yet clear whether high or low PEDF serum level accelerates the development of these diseases. Perhaps the association between PEDF and certain diseases is dependent on different pathogenic factors, pathogenesis, receptors, and even individual variation.

The anti-atherosclerosis application of PEDF is debatable. At present, PEDF is believed to be the most potent inhibitor of angiogenesis. It exerts an anti-angiogenesis effect by causing apoptosis of endothelial cells and by disrupting the balance between pro- and anti-angiogenic factors [10]. Thus, it is conceivable that the local downregulation of PEDF in ischemic lesions of the heart could promote angiogenesis and neovascularization, contributing to increased perfusion of the injured myocardium [10]. In turn, local excess of PEDF in ischemic lesions might suppress inflammation, oxidative stress, and angiogenesis, and further balance the destabilization and rupture of plaques, inhibiting subsequent thrombus formation.

PEDF was originally recognized for its neurotrophic effects [43, 44]. With in-depth studies, mounting evidence has shown that PEDF can exert anti-angiogenesis, anti-inflammation, anti-oxidization, anti-thrombosis, anti-tumor, and vascular protection effects, among others [5, 10, 25, 45, 46]. These properties have increased the importance of PEDF in the area of cardiovascular medicine. Endothelial dysfunction, inflammation, oxidization and angiogenesis are vital events in the development and progression of atherosclerosis; therefore, PEDF has promising therapeutic potential for the treatment of atherosclerosis [10].

## PEDF and atherosclerosis

**Fig. 2:**
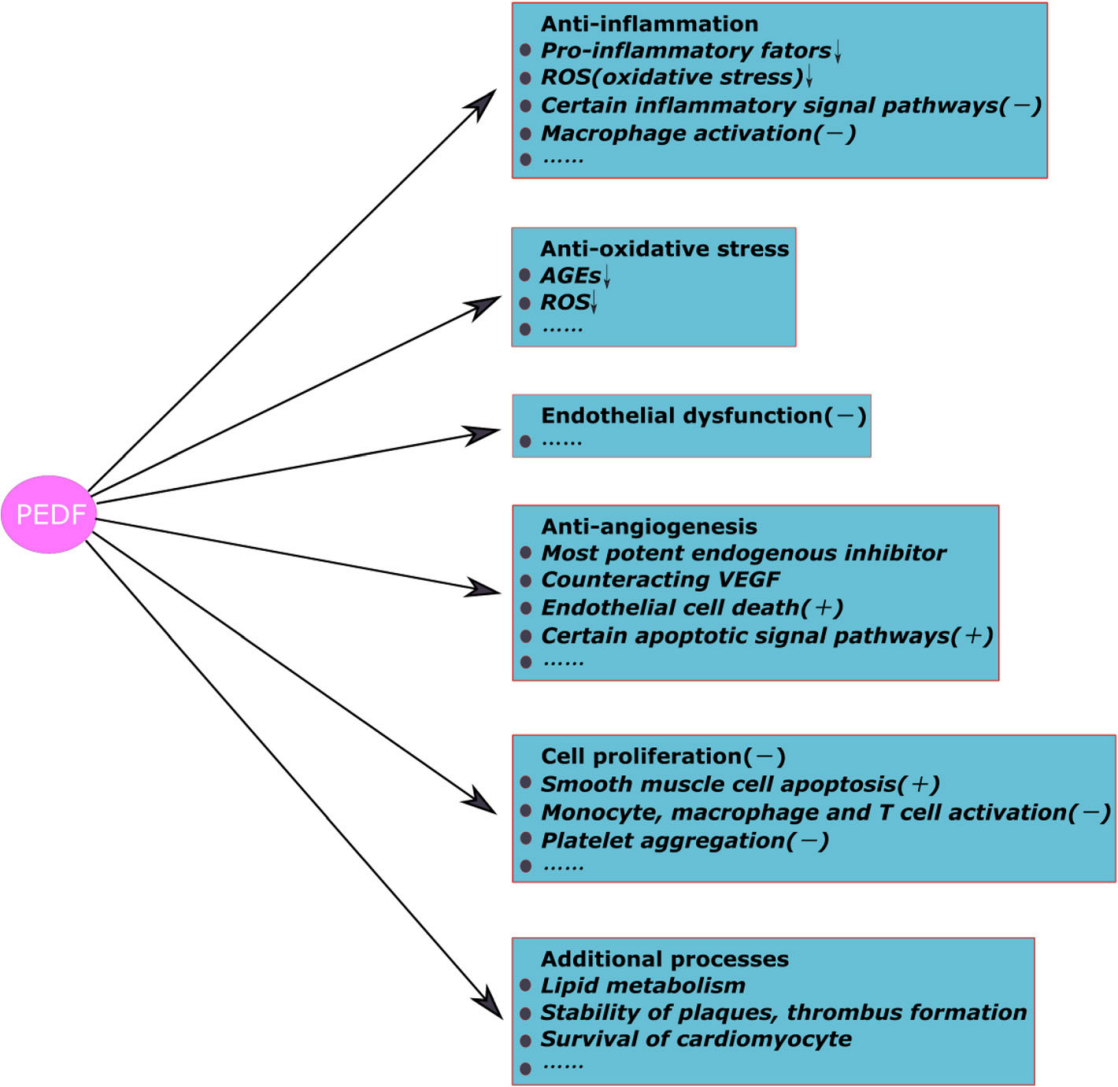
The putative effects of PEDF on atherosclerosis progression. From a physiological view, PEDF can alleviate the development of atherosclerosis based on its anti-inflammatory, anti-oxidant, anti-angiogenic and anti-thrombogenic properties. The underlying mechanisms of PEDF are listed that form a concerted set of activities to protect health against atherosclerosis. PEDF, pigment epithelium-derived factor

The exposure of vascular cells to CVD risk factors, such as lipids, chronic inflammation, hypertension, diabetes, and stress, is important in the development of atherosclerosis [3]. As a result, these pathogenic factors drive endothelial dysfunction. Vascular permeability is increased, driving lipid infiltration and monocyte accumulation and adhesion. Cholesterol accumulation in the vessel wall, in particular low-density lipoprotein (LDL), has a central role in atherogenesis [47]. LDLs that deposit in the intima can be oxidized or be acted upon by enzymes [48]. These modified LDLs, in turn, can stimulate pro-inflammatory factors, including monocyte chemotactic factors, and enhance inflammation [47, 49]. Once monocytes transmigrate and reach the subendothelium, they can differentiate into macrophages, engulf modified lipids and convert into foam cells [50]. Meanwhile, VSMCs migrate to the intima where they produce ECM and induce the formation of fibrous caps [51]. The progression of atheroma, loss of VSMCs, apoptosis of foam cells and the release of active metalloproteases can thin the fibrous cap, increasing the susceptibility of plaques to rupture. Ruptured plaques induce severe stenosis or thrombosis in arteries, which leads to regional ischemic damage, such as acute myocardial infarction (AMI) and even death [52]. From a clinical perspective, PEDF may be a factor directly associated with atherosclerosis, and circulating level of PEDF might act as a biochemical marker of atherosclerosis [18, 53]. From a physiological view, PEDF can play a critical role in atherosclerosis processes based on its anti-inflammatory, anti-oxidant, anti-angiogenic and anti-thrombogenic properties. The following will discuss the underlying anti-atherosclerosis mechanisms of PEDF (Fig. 2).

### Endothelial dysfunction

ECs are essential for maintaining the physiological functions of the cardiovascular system. Endothelial injury or dysfunction has been proposed to be the first step in the initiation of atherosclerosis [54–56]. The presence of severe endothelial dysfunction in CAD patients has been revealed to be a predictor of cardiac death, myocardial infarction (MI) and revascularization [57]. Additionally, endothelial dysfunction is a predictor of adverse outcome in patients after ACS [58–60]. Impaired ECs have been shown to stimulate adhesion molecule expression, release chemotactic factors, increase membrane permeability and enhance the infiltration of LDL into the arterial intima. Furthermore, the subsequent oxidation of LDL exerts cytotoxic effects directly on ECs and promotes the expression of pro-inflammatory cytokines and ROS, which can induce further endothelial dysfunction [61]. Ox-LDL is postulated to be a primary stimulus for monocyte-endothelial interactions. In short, ox-LDL is crucial in endothelial dysfunction and endothelial dysfunction could lead to decreased expression of anti-inflammatory mediators, plaque destabilization [62] and reduced anti-thrombotic tendencies, thereby allowing thrombus formation [63].

PEDF is a multifunctional protein with anti-inflammatory, anti-oxidant, antithrombotic and vascular protective properties. ECs can secret PEDF, and this secretion is critical to suppress the proliferation and migration of VSMCs after balloon injury [32]. There is evidence indicating that PEDF impedes cytokine-, growth factor- and advanced glycation end product (AGE)-induced EC damage [5, 64]. Yamagishi S and colleagues have demonstrated that PEDF could inhibit Ang-II-induced EC activation by suppressing NADPH-oxidase-mediated ROS generation, and that PEDF may play a protective role in the development and progression of atherosclerosis [65]. Meanwhile, PEDF could block tumor necrosis factor (TNF)-induced EC activation through its anti-oxidative properties [17]. In agreement with these findings, we found that an apolipoprotein A-I mimetic peptide, D-4F, effectively reduced ox-LDL-induced endothelial injury by upregulating PEDF [23], suggesting that PEDF may protect ECs against damage. Similarly, our latest data shows that PEDF may mitigate endothelial injury by suppressing the Wnt/β-catenin pathway [24]. In addition to the direct effects of PEDF on ECs, this compound could play an important role in protecting ECs via its diverse properties, such as its anti-inflammatory and anti-oxidant capabilities. As mentioned above, inflammation and oxidative stress can impact endothelial dysfunction and elicit EC damage. Conversely, endothelial injury or dysfunction upregulates the expression of inflammatory factors and promotes the deposition and oxidization of LDL in the intima. Hence, PEDF, with anti-inflammation and anti-oxidation properties, could limit the interaction between inflammation, oxidative stress and endothelial dysfunction.

### Inflammation

Atherosclerosis is a complex chronic disease which develops in the arterial wall, and is driven by various risk factors that lead to excessive inflammatory reactions [66]. The inflammatory response is recognized to be pivotal throughout all the steps of atherosclerosis, such as endothelial dysfunction, the formation of fatty streaks, and unstable plaque rupture [67, 68]. Endothelial dysfunction and lipoprotein accumulation in the intima have been reported to be the earliest events in atherosclerosis with which inflammation is related. Pro-inflammatory factors can directly injury ECs and elicit endothelial dysfunction. In turn, damaged ECs promote membrane permeability and the infiltration of LDL into the arterial intima. Subsequently, LDL is oxidized or modified by AGEs or inflammatory cytokines. Ox-LDL and modified LDL can enhance monocyte-endothelium adhesion, VSMC migration and increased expression of pro-inflammatory cytokines. An imbalance between anti-inflammatory mechanisms and pro-inflammatory factors will result in atherosclerotic progression. Therefore, inflammation is a key feature of the atherosclerotic process [69].

PEDF is an endogenous anti-inflammatory factor, which may have a protective role in atherosclerosis by inhibiting the proliferative inflammatory response to damage [70]. Recent research has found that PEDF could inhibit inflammation in vivo by suppressing the Ang II-induced increased cellular ROS concentration via regulation of the nuclear factor-κB (NF-κB) signal pathway [65]. PEDF could inhibit the macrophage activation elicited by lipopolysaccharides and induce macrophage apoptosis, which demonstrates the potential ability of PEDF to attenuate macrophage-implicated inflammatory activities in diabetic retinopathy [71, 72]. Additionally, PEDF was reported to inhibit the expression of pro-inflammatory factors, including IL-6 [17], vascular endothelial growth factor (VEGF), TNF-α and intercellular adhesion molecule-1 (ICAM-1) [70]. One study concluded that the pro-inflammatory effects of heavily oxidized glycated LDL (HOG-LDL) on retinal pericytes were significantly mitigated by PEDF [73]. In addition, the Wnt/β-catenin pathway is closely related to inflammation, primarily by a variety of important inflammatory factors [74–76]. Besides, there is evidence suggesting that PEDF may reduce inflammation and oxidative stress by inhibition of the Wnt pathway in unilateral ureteral obstruction kidneys [77]. Similar to these results, our team has demonstrated that ox-LDL-induced human umbilical vein endothelial cell (HUVEC) injury and activation of the Wnt/β-catenin pathway were able to be inhibited by PEDF [24]. This effect was, perhaps, partially due to the inhibition of inflammation. Nevertheless, further work is needed to fully elucidate the impacts of PEDF.

### Oxidative stress

Over time, the accumulated evidence from mechanistic studies has indicated the significant role oxidation plays in atherosclerosis pathogenesis. These findings show that oxidative stress can induce the oxidization or modification of lipoproteins, injure vascular ECs, promote the activation of monocytes, macrophages, T cells and platelets, cause vascular inflammation, and increase the proliferation of VSMCs [78, 79]. Evidence has suggested that induction of ox-LDL, the first event in atherosclerosis, activates intracellular oxidative stress [80, 81]. Oxidative stress, consequently, is believed to play a major role in atherosclerosis.

It has been established that PEDF has anti-oxidative properties, and recent advances have been made to identify the detailed inhibitory effects of PEDF on oxidative stress. Previous studies have indicated that PEDF could suppress occlusive thrombus formation by inhibiting platelet activation and aggregation through its anti-oxidative effect [16]. PEDF was shown to suppress NADPH oxidase activity, which is a molecular target for the anti-oxidative properties of PEDF [10]. It is known that AGEs increase oxidative stress generation, and that PEDF alleviates the effects of AGEs [82, 83]. In addition, Zhang SX and coworkers demonstrated that PEDF effectively alleviated HOG-LDL-induced ROS generation via the upregulation of superoxide dismutase 1 (SOD1) [73]. Our group has also studied PEDF anti-oxidation. Initially, we revealed that ox-LDL led to the downregulation of PEDF in HUVECs, which may have been triggered by the ox-LDL-induced promotion of ROS [22]. Further work by our group has shown that D-4F could mitigate the oxidative stress and endothelial injury by upregulating PEDF, and that PEDF attenuates endothelial injury by blocking the Wnt/β-catenin pathway, subsequently ameliorating oxidative stress [24]. These results reveal that PEDF may protect ECs from cytotoxicity and injury via reducing oxidative stress, which may involve certain signal pathways. However, the exact mechanism of oxidative stress is controversial and further research is necessary. Currently, our team is making great efforts in understanding the association between PEDF and oxidative stress by exploring the effect of PEDF on foam cells.

### Angiogenesis

Angiogenesis is characterized by the growth of new blood vessels from preexisting vessels [84] and is a pathological hallmark of chronic vascular disease, such as atherosclerosis and cancer. Similar to endothelial injury, angiogenesis is known to be essential for atherosclerotic plaque development and instability and is a feature of unstable plaques [85, 86]. Plaque rupture or bleeding is a crucial cause of severe acute cardiovascular events and other complications induced by atherosclerosis, and plaque stability is closely related to the density of neovessels in the lesion [9, 87]. This has been corroborated by a study from Dunmore BJ et al., which has shown that VEGF, a promoter of angiogenesis, is positively correlated with the destabilization of carotid artery plaque [88]. Moreover, it has been shown that an increasing severity in atherosclerosis corresponded to an increase in the incidence of neovascularization from 31 to 100%, and the instability of new vascular structures, along with the deficiencies in the basement membrane and pericytes, gave rise to plaque instability [89]. Additionally, research has shown that recombinant endostatin is able to inhibit the growth of neovascularization, reduce the area of atherosclerotic plaque and ameliorate the development of new plaques [90]. Neovascularization can disappear with plaque regression induced by a lowering of cholesterol in animals [85, 91]. Notably, angiogenesis is critical in advanced atherosclerosis.

In contrast to VEGF, PEDF is an endogenous inhibitor of pathological angiogenesis, which potently and specifically suppresses pathogenic neovessel growth without injuring mature and preexisting vessels [92, 93]. There has been much speculation regarding the anti-angiogenesis capabilities of PEDF [94]. Initially, Dawson found that PEDF was a more potent angiogenesis inhibitor than angiostatin, a well-studied angiogenesis inhibitor [25]. PEDF is believed to be a far more potent anti-angiogenesis compound than any other known endogenous factors [95]. Notably, PEDF is able to counteract the effects of VEGF. Many studies, which include both in vitro and in vivo experiments, have revealed that PEDF may suppress angiogenesis by exerting a concentration-dependent effect upon VEGF [96]. Mejias M and associates have found that PEDF upregulation might reflect a compensatory mechanism aimed at alleviating pathological VEGF-induced angiogenesis [93], which is in agreement with other studies [97–99]. In other words, angiogenesis depends on a balance between PEDF and VEGF. Several groups contend that the mechanism of PEDF-inhibited new vessel growth might be involved in endothelial cell death through the activation of different pathways, including the Fas/FasL death pathway [100], the JUN N-terminal kinase (JNK) pathway [101] and FLICE-like inhibitory protein (FLIP) [102]. However, further studies are required to fully elucidate the anti-angiogenic mechanisms of PEDF. The general anti-angiogenic property of PEDF has made it a promising therapeutic target for mitigating the progression of angiogenic diseases such as CVD [10].

### Cell proliferation

Previous studies have suggested that the atherosclerotic plaque develops due to the proliferation of endothelial cells, smooth muscle cells, monocytes, macrophages and inflammatory cells [103]. Endothelial cells activation and the subsequently induced inflammatory reaction are critical in the development of atherosclerosis. One study has reported that the smooth muscle cells in the intima can easily proliferate and synthesize ECM and cytokines, thereby promoting atherosclerosis [104]. Increased proliferation of macrophages in the intima can accelerate the formation of early atherosclerotic plaques [105]. The presence of coronary restenosis confirms the clonal proliferation of vascular wall myointimal cells after angioplasty or stent implantation [106]. Additionally, enhanced cell growth, particularly of inflammatory cells, was found to be related to oxidative stress and inflammation in atherosclerotic lesions.

Accumulating evidence supports the idea that PEDF inhibits the proliferation of several types of cells and triggers cell apoptosis. Yamagishi S and colleagues have indicated that PEDF inhibits angiotensin II-induced smooth muscle cell proliferation due to its anti-oxidative properties [107]. In addition to affecting proliferation, the results of in vitro experiments have shown that PEDF could suppress smooth muscle cell migration, monocyte, macrophage and T cell activation, and platelet aggregation [5, 64]. Moreover, PEDF was found to promote cell apoptosis. Volpert OV found that PEDF increased FasL expression and subsequently induced apoptosis via caspase-8 [100]. PEDF directly or indirectly induces tumor cell apoptosis [95], such as melanoma [108] and glioma cell [109]. However, it has recently been found that PEDF could upregulate FasL expression and facilitate ECs death [100, 110], which is not consistent with our finding that PEDF might reduce endothelial injury [24]. Regarding this issue, Tombran-Tink J and Barnstable CJ have stated that the involvement of PEDF in events that can give rise to both cell survival and cell death lies in the PEDF-activated signaling cascades, suggesting a potential dual activity of PEDF [43]. The mechanisms pertaining to this dual property need to be further discussed.

### Additional processes in atherosclerosis

There are many theories regarding the pathogenesis of atherosclerosis. In addition to those mentioned above, theories relating to the mechanisms of atherosclerosis, including lipid accumulation and infiltration, VSMC mutation, thrombosis and monocyte-macrophage interaction are under investigation. These relevant mechanistic theories require further research. Specifically, PEDF is thought to be correlated with these processes.

Lipids undoubtedly play an essential role in atherosclerosis. PEDF has been identified as a potential regulator of lipid metabolism [111], and loss of PEDF resulted in the accumulation of lipids in ethanol-induced hepatic steatosis [112]. Notari L proposed that ATGL may be a receptor for PEDF [38]. PEDF was found to reduce hepatocyte triglyceride content through its interaction with ATGL in vitro [113]. Furthermore, there are relevant clinical data indicating that serum PEDF level may be related to MeS and diabetes [40, 99, 114]. In turn, lipids are able to regulate the expression of PEDF. Yin L and coworkers demonstrated that ox-LDL could upregulate the VEGF: PEDF ratio in human retinal pigment epithelial cells [115]. Our group has revealed that ox-LDL downregulates PEDF by increasing ox-LDL-induced intracellular ROS [22].

Advanced atherosclerosis is characterized by plaque rupture and thrombosis. The unstable plaque with chronic inflammation is a precursor for adverse cardiac outcomes. Wen H and colleagues have revealed that PEDF improves the stability of atherosclerotic plaques by ameliorating the macrophage inflammation response, which is closely associated with peroxisome proliferator-activated receptor γ (PPAR-γ) and downstream mitogen-activated protein kinase (MAPK) [116]. There is evidence suggesting that PEDF can inhibit occlusive thrombus formation by blocking platelet activation and aggregation [16], and it may prevent atherothrombosis in patients with diabetes mellitus by inhibiting the CD40-CD40L axis [117, 118]. PEDF may instigate macrophage apoptosis and necrosis through the signaling of PPAR-γ, reducing the accumulation and infiltration of macrophages in the lesion area, which increases plaque stability [72].

Interestingly, PEDF has been found to be involved in the survival of cardiomyocytes. Research shown that a reduction in both adenosine triphosphate (ATP) production and expenditure, controlled by PEDF, can reduce cardiomyocyte energy failure and increase its energy reserves, thereby prolonging cardiomyocyte activity during oxygen-glucose deprivation which is associated with an AMP-activated protein kinase (AMPK)-dependent degradation pathway [119]. These data demonstrate the protective effect of PEDF against hypoxia in cardiomyocytes and show that PEDF may exert this function in atherosclerosis-driven ischemic heart disease. Furthermore, Gao X and assistants revealed that PEDF and its functional PEDF-derived peptide 44mer may protect cardiomyocytes against apoptosis and necroptosis under hypoxic conditions via an anti-oxidative effect [120].

## Conclusions

PEDF is a multifaceted compound that can impact atherosclerosis through impacting various pathological responses, including endothelial dysfunction, inflammation, oxidative stress, angiogenesis, cell proliferation and other processes. Current research has also reported on the effective potential of this versatile protein against other diseases, such as eye disorders and cancer. Our group has revealed an association of PEDF with ACS and CAD, and we have explored the role of PEDF in ox-LDL-induced endothelial injury. However, considerably more work is required to fully understand the beneficial effects of PEDF on CVD, particularly on atherosclerosis. First, it is necessary to elucidate how PEDF, either directly or indirectly, impacts each component of atherosclerosis. Second, further research is required to discover the essential domain of PEDF to which the particular effects of the polypeptide are attributed. Third, receptors for PEDF are of great interest in atherosclerotic progression and cell biology. Thus, more extensive studies are needed to clarify the mechanisms of the biochemical pathways that PEDF impacts and the PEDF receptor signaling cascades. In conclusion, due to its diverse effects, PEDF is becoming a promising novel therapeutic agent in the fight against atherosclerosis, without compilations of resistance and toxicity. Although there are many in vitro and in vivo studies indicating the benefits of PEDF, the full potential of this compound requires further clinical exploration.

## Abbreviations

ACS: Acute coronary syndrome
AGE: Advanced glycation end product
AMI: Acute myocardial infarction
AMPK: AMP-activated protein kinase
ATGL: Adipose triglyceride lipase
ATP: Adenosine triphosphate
BNP: Brain natriuretic peptide
CAD: Coronary artery disease
CVD: Cardiovascular disease
EC: Endothelial cell
ECM: Extracellular matrix
FasL: Fas ligand
FasR: Fas receptor
FLIP: FLICE-like inhibitory protein
HF: Heart failure
HOG-LDL: Heavily oxidized glycated LDL
HUVEC: Human umbilical vein endothelial cell
ICAM: Intercellular adhesion molecule
JNK: JUN N-terminal kinase
LDL: Low density lipoprotein
LR: Laminin receptor
LRP: Low density lipoprotein related protein
MAPK: Mitogen-activated protein kinase
MetS: Metabolic syndrome
MI: Myocardial infarction
NF-κB: Nuclear factor-κB
Ox-LDL: oxidized-low density lipoprotein
PEDF: Pigment epithelium-derived factor
PLA_2_: Phospholipase A_2_
PPAR-γ: Peroxisome proliferator-activated receptor γ
ROS: Reactive oxygen species
RPE: Retinal pigment epithelium
SERPIN: Serine protease inhibitor
SNP: Single nucleotide polymorphism
SOD: Superoxide dismutase
TNF: Tumor necrosis factor
VEGF: Vascular endothelial growth factor
VSMC: Vascular smooth muscle cell
